# A Mouse Periodontitis Model With Humanized Oral Bacterial Community

**DOI:** 10.3389/fcimb.2022.842845

**Published:** 2022-02-22

**Authors:** Lan Bai, Bo-Yan Chen, Yan Liu, Wu-Chang Zhang, Sheng-Zhong Duan

**Affiliations:** ^1^ Department of Periodontology, Shanghai Ninth People’s Hospital, Shanghai Jiao Tong University School of Medicine, Shanghai, China; ^2^ Laboratory of Oral Microbiota and Systemic Diseases, Shanghai Ninth People’s Hospital, College of Stomatology, Shanghai Jiao Tong University School of Medicine, Shanghai, China; ^3^ National Center for Stomatology, National Clinical Research Center for Oral Diseases, Shanghai Key Laboratory of Stomatology, Shanghai, China

**Keywords:** periodontitis, mouse model, subgingival plaque, saliva, 16S rRNA sequencing

## Abstract

Increasing evidence suggests that periodontitis, characterized by oral dysbiosis, is a critical player in the progression of multiple systemic diseases in humans. However, there is still a lack of a proper mouse model of periodontitis with the colonization of human periodontitis-associated bacteria. We here established a new mouse periodontitis model by combining ligation of the second molars with application of subgingival plaques from periodontitis patients. Using 16S rRNA gene sequencing and Taxonomic classification, we found that human periodontitis-associated bacteria efficiently colonized in the mouse model and were enriched in both ligature silk and mouse saliva. Furthermore, the well-recognized periodontal pathogens including *Porphyromonas gingivalis*, *Fusobacterium nucleatum*, *Prevotella intermedia*, and *Tannerella forsythia* were enriched in the new model, but not in ligature-induced periodontitis model or Sham mice. The human periodontitis-associated bacteria potently aggravated mouse periodontitis, as demonstrated by more severe bone resorption and higher expression of inflammatory and osteoclastogenesis genes. In summary, the new mouse periodontitis model paves the way for studying human periodontitis-associated bacteria in oral diseases and systemic diseases.

## Introduction

Human periodontitis-associated bacteria cause local destruction of periodontal tissue and are also tightly linked to the progression of multiple systemic diseases. Periodontitis (PD) is a biofilm-induced chronic inflammatory disease of the tooth-supporting tissues, which destroys gingiva and alveolar bone, eventually causes teeth loss (Brown LJ and Löe, 1989). The number of new periodontitis cases is about 701/100,000 each year, and periodontitis becomes the 6^th^ most prevalent diseases all over the world (Kassebaum et al., 2014). Some studies suggest that the dysbiosis of oral microecology not only causes periodontitis, but affects other organs (Hajishengallis, 2015). Accumulating evidence has linked periodontal disease with cardiovascular diseases, metabolic diseases, and cancers (Michaud et al., 2017; Polak and Shapira, 2018; Sanz et al., 2020). Although increasing attentions are paid on the human oral dysbiosis during understanding the pathogenesis of systemic diseases, there is no proper mouse model of periodontitis with the colonization of human periodontitis-associated microbiota.

The microbiota of the human oral cavity consists of a myriad of bacterial species, which normally exist in commensal harmony with the host (Mysak et al., 2014). The dominant flora in oral cavity of periodontitis patients is massively different from those in healthy people. The current concept of the etiology of periodontitis is that bacterial components of the biofilm initiate the inflammatory cascade, including infiltration of immune cells and production of inflammatory mediators in the periodontal tissue (Bage et al., 2010). It has been demonstrated that *Porphyromonas gingivalis*, *Tannerella forsythia*, and *Treponema denticola* are closely related to periodontitis progression (Socransky et al., 1998), and that *Synergistes*, *Filifactor*, and *Mycoplasma* also take part in periodontal disease (Shi et al., 2015). Besides causing tooth loss, periodontal pathogens have a systemic impact through a variety of mechanisms. These include bacteremia caused by the translocation of periodontal pathogens into the systemic circulation and endotoxemias due to the lipopolysaccharides of the periodontal-pathogenic bacteria.

For particular purposes, some mouse periodontitis models including inoculation of well recognized periodontal pathogens, LPS injection, and ligation of molars have been constructed (Blasco-Baque et al., 2017; Costa et al., 2021). Ligation of molars is a classical mouse periodontitis model, making great contributions to the elucidation of mechanisms of periodontitis. However, these models cannot meet the currently increasing demands in the field, such as screening the human periodontal pathogens critical for systemic diseases. Previous studies suggested that *Bifidobacteria*, which was common bacteria in human oral microflora, was not detected in mouse oral cavity (Mackie et al., 1999). Furthermore, the dominancy of *Staphylococcus* species in the mouse was not found in the human oral microflora (Paster et al., 2006; Keijser et al., 2008). Moreover, even within the common bacterial orders, the actual families, and species were often different between mice and humans (Hasegawa et al., 2010). Therefore, the current experimental animal and *in vivo* models cannot fully summarize the human situation, despite that these models can effectively address particular aspects of the disease.

In this study, we aimed to establish a mouse model of periodontitis with colonization of human periodontitis-associated oral microbiota. We combined the ligature-induced mouse periodontitis (LIP) with transplantation of subgingival plaque from periodontitis patients. Firstly, we used 5-0 silk suture ligaturing mouse second molars. We then transplanted subgingival plaque of periodontitis patients on the silk suture. Finally, we analyzed the alveolar bone resorption and the composition of microbiota. The results suggested that we successfully established a mouse periodontitis model, whose composition of oral bacteria was similar to periodontitis patients.

## Methods

### Subject Recruitment

This study was approved by the Ethics Committee of Shanghai Ninth People’s Hospital, Shanghai Jiao Tong University School of Medicine. Informed consent was signed by all subjects before enrollment. All medical data were collected according to clinical standard procedures.

The clinical periodontal examination was performed by a single trained examiner before the collection of subgingival plaque (SP). Severe PD was diagnosed used the following criteria (Timmerman et al., 1998): 1) gum bleeding within 15 seconds after probing; 2) at least one site with periodontal pocket depth > 5mm; 3) at least one site with attachment loss > 4mm. Patients who had taken any antibiotic or probiotic, smoke, or had undergone periodontal therapy in the previous 6 months were excluded.

### Sample Collection

SP were collected from six sites showing the deepest probing depth of each patient. All SP samples were stored in 20% glycerin at -80°C until further processing.

### Animals

Male C57/B6J adult mice (8~10w) were used for experiments. And all experiments were repeated more than three times. The mice were randomly divided into three groups: Sham group (n=3), LIP group (n=3), and LIP+SP group (n=6). LIP was established by 5/0 silk suture around the bilateral maxillary second molars of mice (Abe and Hajishengallis, 2013). All SP samples were mixed and centrifuged at 5,000g for 5 minutes. Then, precipitate was resuspended in sterile 20% glycerin and was divided into tubes (the number of tubes was same as the quantity of patients). Again, centrifuged at 5,000g for 5 minutes, the pellets were resuspended in 1 ml sterile 2% carboxymethylcellulose (CMC). Application of SP (100μl per mouse) or 2% CMC to mouse teeth began on the next day, once every two days for 14 days (totally 7 times). Oral swabs of each mouse were collected one day before sacrificing. Four weeks after ligation, mice were euthanized, silk sutures, gingiva, and maxilla were collected. Mice in Sham group were ligated for 4 hours before being sacrificed. Mice were excluded if they died after the operation.

The animal experiments were approved by the Institutional Review and Ethics Board of Ninth People’s Hospital, Shanghai Jiao Tong University School of Medicine.

### Micro-CT Analysis

Maxillary bone and teeth were collected and fixed in 4% phosphate-buffered paraformaldehyde for 72 h. Then, the maxillae were processed for micro-computed tomography (CT) scanning using Bruker SkyScan 1176 (SkyScan) at a voxel resolution of 9 μm. Measurements were performed on the lingual sides of the maxillary second molar. The distance from the cementoenamel junction (CEJ) to the alveolar bone crest (ABC) was measured. Bone mineral density (BMD) and bone volume/total volume (BV/TV) were also measured.

### Histology

The maxillae were fixed in 4% phosphate-buffered paraformaldehyde for 72 h, then decalcified in 10% EDTA solution for 4 weeks. The EDTA solution was changed daily until decalcified. Maxillae were embedded in paraffin and cut into 5μm sections, which were then prepared for hematoxylin and eosin (HE) staining. HE staining was conducted according to routine protocols. The staining was observed under a microscope and photographed with 50X and 100X lens.

### Quantitative RT-PCR

Gingiva RNA was extracted using Trizol (Life Technologies/Thermo Fisher Scientific) and cDNA was synthesized using reverse transcription kits (Takara, Shiga, Japan). QRT-PCR was performed with a SYBR Green PCR Master Mix (Takara) on a LightCycler480II system (Roche Diagnosticscs, Indianapolis, IN, USA). The sequences for the primers are listed. Il1β forward primer, 5′-GAAATGCCACCTTTTGACAGTG-3′, reverse primer, 5′-TGGATGCTCTCATCAGGACAG-3′. Il17a forward primer, TTTAACTCCCTTGGCGCAAAA, reverse primer, CTTTCCCTCCGCATTGACAC. Rankl forward primer, CAGCATCGCTCTGTTCCTGTA, reverse primer, CTGCGTTTTCATGGAGTCTCA.

### High-Throughput Sequencing and Processing

Silk sutures, saliva swabs, and SP were used for high-throughput Sequencing. The genomic DNA was extracted and the bacteria was identified by 16S ribosomal RNA (rRNA) sequencing. PCR amplification of the nearly full-length bacterial 16S rRNA genes was performed using the forward primer 27F 5’-AGAGTTTGATCMTGGCTCAG-3’ and the reverse primer 1492R 5’-ACCTTGTTACGACTT-3’. The PCR products were quantified with PicoGreen dsDNA Assay Kit (Invitrogen, Carlsbad, USA) and sequenced on PacBio Sequel platform at Shanghai Personal Biotechnology Co., Ltd (Shanghai, China).

Procession of the sequencing data was performed on QIIME2 platform. Analysis of sequencing data was based on amplicon sequence variants (ASVs) (Bokulich et al., 2018). After chimera detection, high-quality sequences with 97% similarity were clustered into the same ASV. Classification of ASVs was performed based on the Greengenes Database.

### Data Analysis

Richness and a-diversity were measured by Chao1 and Shannon indices based on the species profiles (Chao, 1984; Shannon, 1948). Beta diversity was visualized using principal coordinate analysis (PCoA) based on the Bray-Curtis distances. Taxa abundances at the species levels were compared among groups by MEGAN (Huson et al., 2011). LEfSe (Linear discriminant analysis effect size) was performed to detect differentially abundant taxa across groups using the default parameters (Segata et al., 2011). Venn diagram was generated to visualize the shared and unique species among groups using R package “Venn Diagram”, based on the occurrence of species across groups regardless of their relative abundance (Zaura et al., 2009).

### Statistics

All data were shown as mean ± SEM. Statistical analysis was performed using Prism 5.0 (GraphPad Software, La Jolla, CA, USA). The differences between means of two experimental groups were analyzed by unpaired Student’s *t* test or non-parametric test. Values of p ≤ 0.05 were considered statistically significant.

## Results

### Human Periodontitis-Causing Bacteria Efficiently Colonize in the Mouse Model

**Figure 1:**
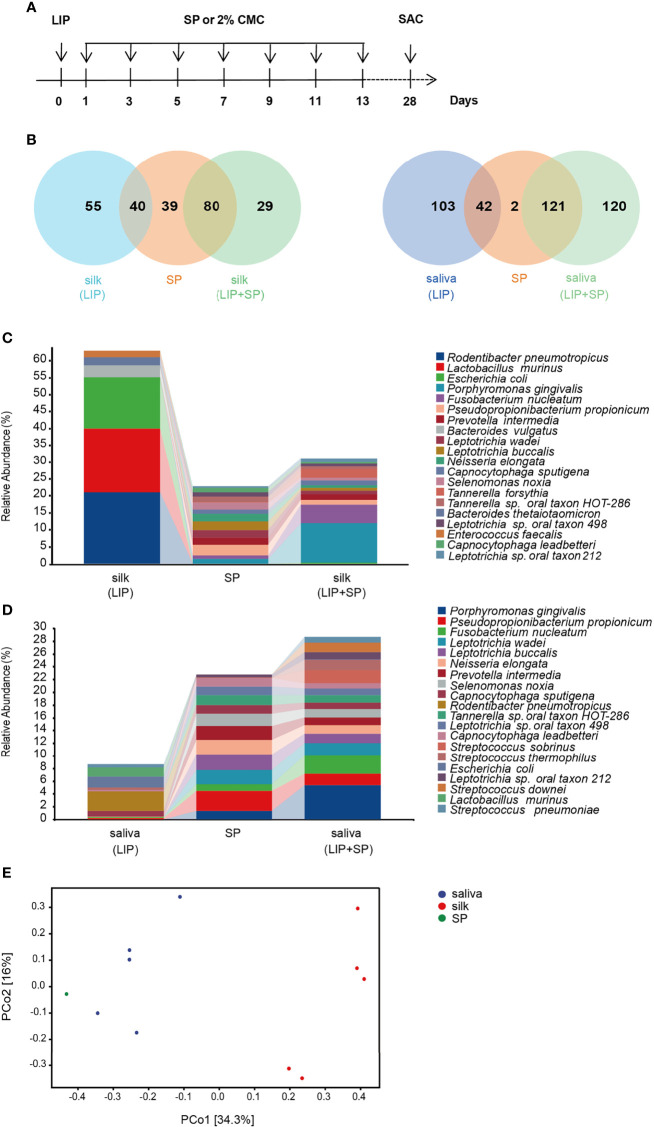
Human periodontitis-causing bacteria efficiently colonize in the mouse model. **(A)** Schematic illustration of experimental design. LIP, ligature-induced periodontitis. SP, subgingival plaques from patients with periodontitis. CMC, carboxymethylcellulose, and SAC, sacrifice. **(B)** The Venn diagrams showing the overlapped and different bacteria species between mouse silk ligature and human subgingival plaque (left), and between human subgingival plaque and mouse saliva (right). **(C)** Taxonomic composition of bacterial community at species level in mouse silk ligature and human subgingival plaque. **(D)** Taxonomic composition of bacterial community at species level in mouse saliva and human subgingival plaque. **(E)** β-diversity of SP and saliva, silk ligature bacteria at species level in LIP + SP group, assessed by principal coordinate analysis (PCoA) based on bray–curtis distance. (n = 5:5).

To establish the mouse model of periodontitis with colonization of human periodontitis-associated oral microbiota, we combined the ligature-induced mouse periodontitis with transplantation of subgingival plaque from periodontitis patients. In detail, we first ligatured the second molar with silk suture in mice, and then applied the bacteria, dissolved in 2% carboxymethylcellulose, on the ligature silk once every other day. The whole period of model construction was 4 weeks (**Figure 1A**). Lastly, 16S rRNA gene sequencing and taxonomic classification were carried out for unbiased measurement of bacterial composition and abundance in human subgingival plaque, mouse saliva and ligature silk.

We first compared the numbers of same bacteria in the ligature silk and saliva as in human subgingival plaque in both LIP and LIP + SP groups. As showed by Venn diagrams, 40 and 80 species of bacteria were shared by the ligature silk and human subgingival plaque in LIP and LIP + SP mice respectively. Meanwhile, 42 and 121 species of bacteria are same in the saliva and subgingival plaque in LIP and LIP + SP mice respectively (**Figure 1B**). Consistently, the analysis of bacterial composition showed similar results. The bacterial compositions in the ligature silk (**Figure 1C**) and saliva (**Figure 1D**) were significantly more comparable to those in human subgingival plaque in LIP + SP mice versus LIP mice. It is interesting that the well-known periodontal pathogenic bacteria including *Porphyromonas gingivalis* (*P.g*), *Fusobacterium nucleatum* (*F.n*), *Prevotella intermedia (P.i)*, and *Tannerella forsythia* (*T.f*) were markedly colonized in LIP + SP mice, but not in LIP mice. Then, we analyzed the sample diversity using principal coordinate analysis (PCoA). In LIP + SP group, the bacterial composition of saliva is more similar to the SP compared with silk sutures (**Figure 1E**). These results cumulatively demonstrated that the transplantation of human subgingival plaque significantly promoted the enrichment of human periodontitis-causing bacteria in mouse periodontitis model.

### Pathogenic Bacteria of Human Periodontitis Are Enriched in the Ligature Silk in the Mouse Model

**Figure 2:**
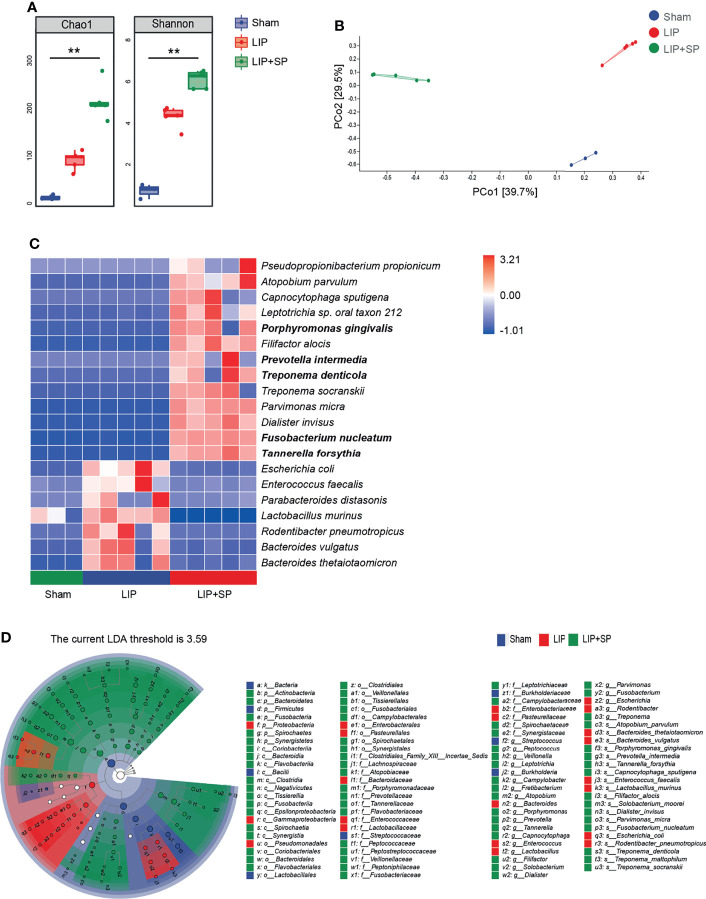
Pathogenic bacteria of human periodontitis are enriched in the ligature silk in the mouse model. Bacterial community of ligature silks from Sham, LIP, and LIP + SP group mice were analyzed using 16S rRNA gene sequencing. **(A)** α-diversity of bacteria on ligature silks assessed by Chao1 and Shannon indices. **(B)** β-diversity of bacteria on ligature silk assessed by principal coordinate analysis (PCoA) based on Bray–Curtis distance of bacteria at species level. **(C)** The heat map of the relative abundance of the top 20 most abundant species of bacteria on ligature silks. **(D)** Taxonomic cladogram of bacteria on ligature silk using LEfSe (LDA = 3.59). The values represent means ± SEM (n = 3:5:5). **P < 0.01.

We further analyzed the change of bacterial composition on ligature silks by sequentially comparing LIP with Sham mice, and LIP + SP with LIP mice. As expected, LIP groups of mice showed significantly higher diversity of subgingival bacteria in comparison with sham mice, and human subgingival plaque transplantation further markedly enlarged the increase of bacterial diversity (**Figure 2A**). PCoA based on Bray-Curtis distance was performed to determine β-diversity (between-sample diversity) of bacteria composition on ligature silks, which demonstrated the potent distinction among Sham, LIP, and LIP + SP groups of mice (**Figure 2B**). Furthermore, the Heatmap of relative abundances of bacteria species showed that thirteen species of bacteria were massively enriched in the LIP + SP group. Some of these bacteria are periodontal pathogens, including *Porphyromonas gingivalis (P.g), Prevotella intermedia (P.i), Treponema denticola (T.d)*, and *Fusobacterium nucleatum (F.n)*, while they were detected at extremely low abundance in Sham and LIP groups (**Figure 2C**). We next employed linear discriminant analysis effect size (LEfSe) to identify taxa that discriminate microbial composition among three groups of mice. Again, it was shown that the periodontitis-causing bacteria species including *P.g, P.i, T.d, F.n* and *T.f* were more enriched in ligature silk of LIP+SP mice versus Sham and LIP mice (**Figure 2D**).

### Pathogenic Bacteria of Human Periodontitis Are Enriched in the Mouse Saliva

**Figure 3:**
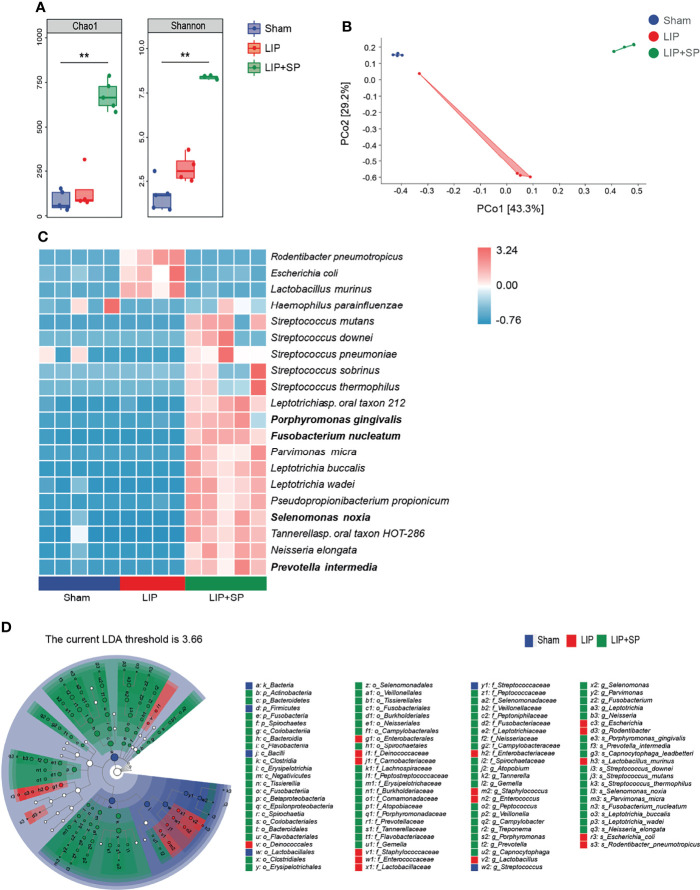
Pathogenic bacteria of human periodontitis are enriched in the mouse saliva. Saliva bacteria from Sham, LIP, and LIP + SP group mice were analyzed using 16S rRNA gene sequencing. **(A)** α-diversity of saliva bacteria assessed by Chao1 and Shannon indices. **(B)** β-diversity of saliva bacteria at species level, assessed by principal coordinate analysis (PCoA) based on Bray–Curtis distance. **(C)** The heat map of the relative abundance of the top 20 most abundant bacteria species in mouse saliva. **(D)** Taxonomic cladogram of saliva bacteria using LEfSe (LDA = 3.65). LDA, Linear Discriminant Analysis. The values represent means ± SEM (n = 5:4:5). **P < 0.01.

By the same strategies, we analyzed the differences of bacteria composition and abundance in saliva among Sham, LIP and LIP + SP groups of mice. The alpha diversity, illustrated by the Chao1 and the Shannon indices, was significantly increased by LIP + SP treatment compared to LIP treatment or no treatment (**Figure 3A**). In the PCoA analysis for β-diversity, the dots of LIP + SP group were far away from the dots of Sham and LIP group, demonstrating the significant divergence in their bacteria composition (**Figure 3B**). We used the Heatmap to show the top 20 most abundant bacteria species in saliva (**Figure 3C**). In LIP + SP group, the compositions of microbial species markedly differed from those in Sham and LIP groups. At the species level, the relative abundances of *F.n*, *P.g*, *P.i*, and *S.n*, which had been shown the close association with periodontal diseases, were sharply increased by human subgingival bacteria transplantation. LEfSe analysis consistently illustrated that these periodontal pathogenic bacteria were significantly enriched in saliva of LIP + SP group mice (**Figure 3D**).

### Human Periodontitis-Associated Bacteria Worsen Periodontitis in Mice

**Figure 4:**
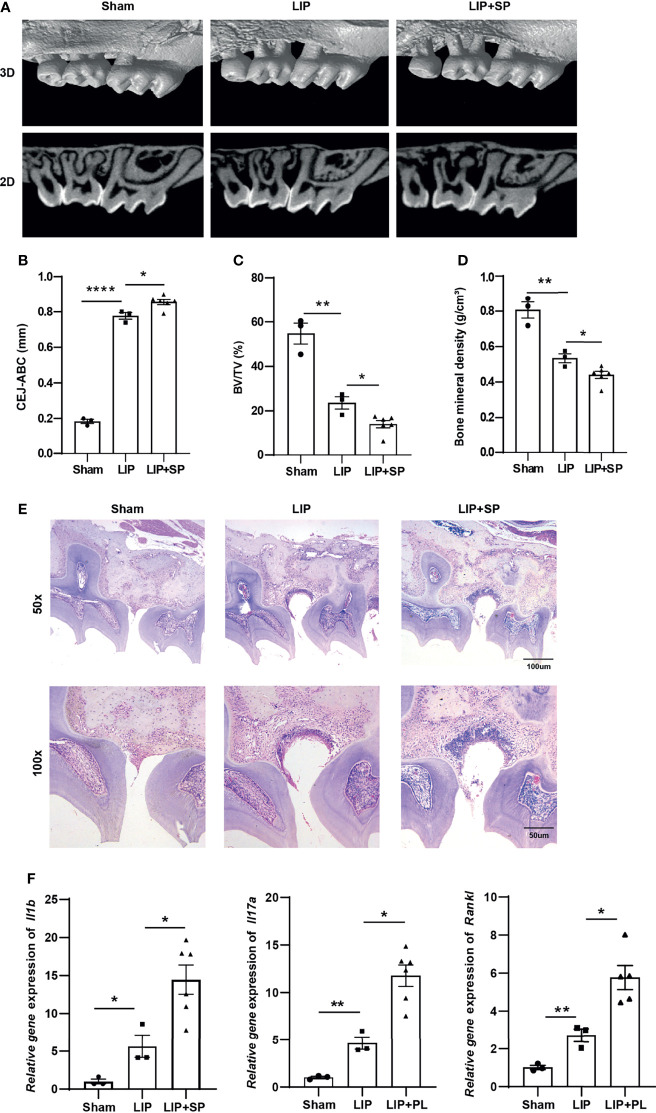
Transplantation of human periodontitis-associated bacteria worsens periodontitis in mice. **(A)** Representative images of Micro–computed tomography scanning of left maxilla from the indicated groups of mice. Both three-dimensional volume (Upper panels) and multiplanar reconstruction (Lower panels) are shown. **(B)** Quantification of the distance from cementoenamel junction (CEJ) to alveolar bone crest (ABC). **(C)** The ratio of bone volume (BV) to total volume (TV) of maxilla surrounding the second molar. **(D)** Bone mineral density (BMD) (g/cm^3^) of maxilla surrounding the second molar. **(E)** Representative hematoxylin–eosin (H&E) staining images of periodontal tissue showing the pathogenic alteration. **(F)** QRT-PCR analyses of inflammatory and osteoclastogenesis gene expression. *Gapdh* was used as internal references. The values represent means ± SEM (n = 3:3:6) from three independent experiments. ns, not significant. *P < 0.05, **P < 0.01, ****P < 0.0001.

To measure effects of the colonized periodontitis-associated bacteria on periodontal tissue in our mouse model, Micro-CT analysis, H&E staining, and real time QPCR assay were performed. The reconstruction images of maxilla showed that LIP caused significant loss of alveolar bone, and the loss was further aggravated by additional treatment of human subgingival plaque (**Figures 4A, B**). As a result, percentage of bone volume (BV) to total volume (TV) was significantly decreased by LIP, and BV/TV was much lower in LIP + SP group versus LIP group (**Figure 4C**). Consistently, there was a significant decline in maxilla bone mineral density (BMD) in both LIP and LIP + SP groups, and the decrease was much greater in LIP + SP group than that in LIP group (**Figure 4D**). In both LIP and LIP + SP groups, hematoxylin–eosin (H&E) staining of maxilla sections showed marked destruction of periodontal tissue around the second molar (**Figure 4E**). In addition, the mRNA levels of inflammatory genes including interleukin-1b (*Il1b*) and interleukin-17a (*Il17*a), and osteoclastogenesis gene, receptor activator of NF-κB ligand (*Rankl*), were substantially higher in the LIP + SP group than those in LIP group. Similarly, the expression of these genes was significantly higher in both periodontitis group versus in Sham group (**Figure 4F**).

## Discussion

Human periodontitis-associated bacteria cause local destruction of periodontal tissue and are also tightly linked to the progression of multiple systemic diseases. However, there is still a lack of proper mouse model of periodontitis with the colonization of human periodontitis-associated microbiota. Here, we created a new mouse model to simulate the clinical situation by transplanting SP on silk sutures, which was used to ligate mouse molars to induce periodontitis. In this model, the composition of mouse periodontal bacteria matched better with that of periodontitis patients.

Previous studies established PD model by silk ligature or specific periodontal bacteria, which induced chronic periodontal disease and systemic inflammation. However, it failed to simulate the original periodontal bacterial composition of PD. The composition of normal oral flora of humans and animals is different (Hasegawa et al., 2010). In the silk suture ligation-induced periodontal model, the main periodontal pathogens are changed, which play a role in the process of periodontitis. This conclusion was supported by in our study that the microbiota composition of the LIP group was totally different from SP. Periodontitis is mainly caused by the imbalance of multiple microbial floras (Socransky et al., 1998). The use of a specific bacterium does not reflect the role of other bacteria in periodontitis, especially the host immune response. Different bacteria are associated with particular function in innate responses and the generation of distinct T-cell subsets (David Jarrossay et al., 2001). For example, Toll-like receptor 2 can recognize *P.g*, *T.f*, *P.i*, and *T.d*. Besides, *Aggregatibacter*, *actinomycetemcomitans*, and *Veillonella parvula* are the pathogens of Toll-like receptor 4 (Cekici et al., 2014). Lastly, periodontitis can affect other systemic diseases (such as cardiovascular diseases, metabolic diseases, and cancers). The possible mechanism includes direct colonization of bacteria on target organs (Costa et al., 2021). The method of using silk sutures to ligate or smear a single bacteria cannot effectively locate the colonized bacteria. As a result, the experimental model *in vivo* is not always appropriate for mimicking clinical settings.

Different types of animals, including mice, rats, dogs, and non-human primates, have been used to establish periodontitis models. However, mice are still the most convenient, cost-effective and versatile models. Advantages of the mice as a model include the considerable background information on their immune system, a wide range of genetically engineered strains (e.g., gene knockouts for key immune receptors or signaling molecules) and availability of high-quality immunochemical and cellular reagents (Graves et al., 2008). Ligation of maxillary second molars in mice is a common periodontitis modeling method. Many articles on periodontitis models have adopted the method of ligating maxillary second molars (Li et al., 2021). Compared with first molars, ligating the second molar is more solid and suture is not easy to slip off. The maxillary third molar is too small and more difficult to operate. In addition, the second molar is adjacent to the first and third molar, where periodontal pathogens can colonize the adjacent teeth.

To our knowledge, this work is the first one to establish a sustained model to simulate the clinical situation. Ligation of second molars combining with patients’ plaques can establish a ‘two hit’ model. This two-hit mouse model of PD has its unique merits. Ligation of molars is not only convenient and time-saving but also facilitates the accumulation of bacteria. In other ligation-induced periodontal models, researchers usually choose two weeks to study how PD affects systemic diseases (Kitamoto et al., 2020). However, these studies might not be representative of the oral health condition and the oral microbiome composition. In this study, we sacrifice mice and harvest samples after ligation for four weeks, which is enough for patients’ bacteria colonizing. In contrast to single periodontal pathogens, patients’ plaques have complex microflora, which is more representative to simulate periodontitis patients’ oral condition.

We observed significant microbial alterations in oral cavity of LIP + SP group mice. Salivary microbiota showed higher richness than that of silk suture of LIP + SP group mice. In comparison with silk sutures, saliva provides larger space, more diverse nutrients, and mobile liquid environment. The different microbiota enriched in the ligation suture and saliva is probably due to the difference of local environment between saliva and ligature silk sutures. Besides, bacteria species of LIP + SP are far more than those of LIP and Sham groups. This is likely because there is more bacterial diversity in subjects with periodontal disease (Abusleme et al., 2013). Furthermore, the dominant pathogens of periodontitis include ‘red complex’ (P. g, T. d, and T. f) and ‘orange complex’ (F. n and P. i, etc.) (Hajishengallis and Lamont, 2012), and the progression of periodontitis is mainly caused by the dominant pathogens. Single periodontal pathogen may not cause disease as expected. Previous study illustrates that P. g is not a potent stand-alone inducer of inflammation. *In vitro* and vivo, P. g often induces contradictory hosts responses. For example, P. g lipopolysaccharide can antagonize toll‐like receptor 4, unlike other highly pro‐inflammatory lipopolysaccharides from most gram‐negative bacteria. Also, in the absence of commensal bacteria, P. g fails to induce periodontitis when used as a mono‐infection in germ‐free mice (Hajishengallis and Lamont, 2012). Therefore, application of SP is a better choice than specific pathogen to induce periodontitis.

## Conclusion

In summary, we established a ‘two-hit’ periodontitis model which combined ligation of mouse molars with subgingival plaques from periodontitis patients. The microbiota composition of silk suture and saliva was similar to that of patients’ subgingival plaques. Additionally, using this animal model, we found that subgingival plaques exacerbated ligation-induced periodontitis and promoted gingiva inflammation.

## Data Availability Statement

The datasets presented in this study can be found in online repositories. The names of the repository/repositories and accession number(s) can be found below: https://www.ncbi.nlm.nih.gov/bioproject/PRJNA793991.

## Ethics Statement

The protocol was approved by the Institutional Review and Ethics Board of Shanghai Ninth People’s Hospital, Shanghai Jiao Tong University School of Medicine. The patients/participants provided their written informed consent to participate in this study. The animal study was reviewed and approved by Institutional Review and Ethics Board of Ninth People’s Hospital, Shanghai Jiao Tong University School of Medicine.

## Author Contributions

S-ZD and W-CZ designed and supervised the project. LB, B-YC, and YL collected the clinical samples, performed the statistical analyses, and wrote the manuscript. S-ZD and W-CZ read and revised the manuscript. All authors contributed to the article and approved the submitted version.

## Funding

This work was supported by grants from the National Natural Science Foundation of China (81991503, 81991500, 81921002) and the Innovative Research Team of High-Level Local Universities in Shanghai (Oral-Gut Ecology and Major Chronic Diseases, SHSMU-ZDCX20212500).

## Conflict of Interest

The authors declare that the research was conducted in the absence of any commercial or financial relationships that could be construed as a potential conflict of interest.

## Publisher’s Note

All claims expressed in this article are solely those of the authors and do not necessarily represent those of their affiliated organizations, or those of the publisher, the editors and the reviewers. Any product that may be evaluated in this article, or claim that may be made by its manufacturer, is not guaranteed or endorsed by the publisher.
